# INSIGHT INTO MEIOTIC DNA END RESECTION: MECHANISMS AND REGULATION

**DOI:** 10.1016/j.dnarep.2025.103886

**Published:** 2025-08-15

**Authors:** Soonjoung Kim, Hasan F Alnaser, Scott Keeney, Hajime Murakami

**Affiliations:** 1 Molecular Biology Program, Memorial Sloan Kettering Cancer Center, New York, New York 10065, USA; 2 Department of Microbiology and Immunology, Institute for Immunology and Immunological Diseases, Yonsei University College of Medicine, Seoul 03722, Korea; 3 Chromosome and Cellular Dynamics Section, Institute of Medical Sciences, University of Aberdeen, Aberdeen, AB25 2ZD, UK; 4 Howard Hughes Medical Institute, Memorial Sloan Kettering Cancer Center, New York, New York 10065, USA

**Keywords:** Meiosis, DNA double-strand break resection, Mre11, Rad50, Nbs1, Xrs2, Exo1

## Abstract

Meiosis generates reproductive cells with a reduced genome complement, with most species using homologous recombination to promote accurate meiotic chromosome segregation and to generate genetic diversity among offspring. A critical step in homologous recombination is DNA end resection, in which DNA double-strand breaks (DSBs) are processed by nucleases to yield the 3’ single-stranded DNA (ssDNA) needed for homology search and strand invasion. DSB resection in nonmeiotic contexts has been extensively studied, but meiotic resection is less well understood. We provide here a review of studies elucidating the mechanism and regulation of resection during meiosis, covering similarities and differences from resection in mitotically dividing cells. The nucleases that carry out resection are discussed, along with resection-modulating factors such as DNA damage signaling and chromatin structure. We focus on the budding yeast *Saccharomyces cerevisiae* and on mouse, for which the most information is currently available, but also describe studies in other species that point to evolutionary conservation or divergence in this key process needed for genome integrity in the germline.

## 1. Introduction

Meiosis is a specialized cell division that occurs in sexually reproducing organisms to produce cells (e.g., spores in yeast; sperm or eggs in animals) with a genome complement that is halved from that of the starting cells. Meiosis accomplishes genome reduction through one round of DNA replication followed by two successive rounds of chromosome segregation. In early stages of meiotic prophase I, programmed DSBs are formed and then repaired through homologous recombination. Meiotic recombination preserves genomic integrity while also increasing genetic diversity [1]. DNA end processing, known as “resection,” is a prerequisite for DSB repair via recombination in all types of cellular contexts, and its regulation has been extensively reviewed [2, 3]. Here, we focus specifically on the mechanisms governing DNA end resection during meiosis.

**Figure 1.**
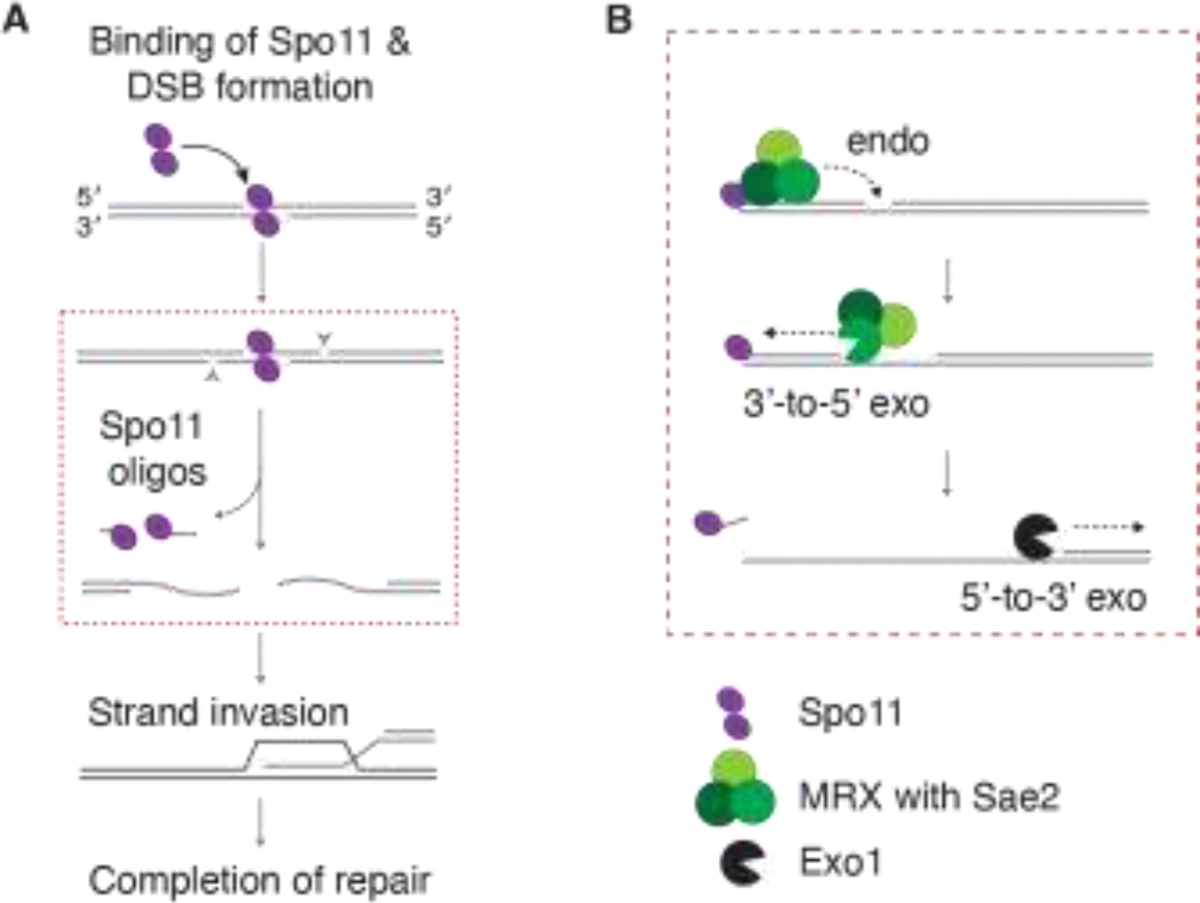
Meiotic DNA end resection in *S. cerevisiae*. (A) Spo11 (purple ellipses) introduces DSBs through a covalent protein-DNA intermediate. Exonucleases access these breaks via nicked sites (gray arrowheads), releasing Spo11-oligo complexes and generating long 3’ ssDNA tails that are used for homology search and strand invasion, completing homologous recombination. (B) The first step (often termed “short-range resection”) in the two-step resection model involves MRX-mediated resection initiation by endonucleolytic nicking near the Spo11 cleavage site, followed by limited 3’-to-5’ digestion. The second step (often referred to as “long-range resection”) involves exonucleolytic extension carried out by Exo1 in the 5’-to-3’ direction. Adapted from [39] under a CC-BY 4.0 license.

Meiotic DSBs are termed ‘programmed’ because they are formed via a developmentally regulated mechanism, in contrast to the more stochastic DSBs that can be caused by DNA-damaging chemicals, radiation, or replication errors and other cellular mishaps. Meiotic DSB formation is controlled regarding number, timing, and positioning [4–7]. The DNA strand breaks are mediated by a dimer of the evolutionarily conserved Spo11 protein, which performs a topoisomerase-like reaction to form a covalent protein-DNA intermediate [8, 9]. The 5’-terminal strands of broken DNA are then efficiently resected by a group of endo- and exonuclease activities, resulting in release of a short DNA fragment attached to Spo11 (Spo11 oligo) and an extended 3’ single-stranded DNA (ssDNA), which is used for homology search, strand invasion and the subsequent completion of recombination (Figure 1A).

Meiotic DSB resection has been most comprehensively studied in budding yeast, which employs a two-step mechanism (Figure 1B). In the first step, the conserved Mre11-Rad50-Xrs2 (MRX) complex, in conjunction with Sae2, cleaves the Spo11-bound strands using the endonuclease activity of Mre11 [9–14] and degrades ssDNA towards the DSB using the 3’-to-5’ exonuclease activity of Mre11 [15]. This step is known as resection initiation or short-range resection [16–18]. In the second step, the more processive 5’-to-3’ exonuclease activity of Exo1 extends the degradation further from the DSB, known as long-range resection.

The terms “short-range” and “long-range” have been used for the steps in yeast meiotic resection to draw parallels to the hand-off from MRX-Sae2 to either Exo1 or Dna2-Sgs1 that occurs during DSB resection in vegetative yeast cells (reviewed in [19]). However, Exo1-mediated resection during yeast meiosis is constrained to considerably shorter distances (less than 1 kb on average in wild type and increased only moderately in repair-deficient mutants [16, 20, 21]) than during mitotic resection (tens of kb or more in repair-deficient mutants), and details of the resection machinery and its regulation are different in meiotic and mitotic resection (see Section 4.1 and Section 7). Moreover, recent studies in mouse spermatocytes indicate that MRN-CtIP (the mammalian ortholog of MRX-Sae2) is responsible for most of the length of resection tracts while EXO1 contributes a smaller, polishing function [20–22]. Thus, while a two-step hand-off from MRX-Sae2 to another nuclease(s) appears to be a common thread across species and between cellular contexts, there are also key differences that may be obscured behind the use of a common terminology that refers only to resection length.

## 2. Advances in measuring meiotic DNA end resection

In many species, meiotic DSBs occur preferentially within defined regions called DSB hotspots [23–26] and are repaired within a tightly regulated timeframe [27]. Hotspot widths average 189 bp in budding yeast [26] and 143 bp in mice [25]. This narrow localization facilitates measurement of the length of resection by various methods (Table 1).

Early studies in *S. cerevisiae* used one-dimensional gel electrophoresis of restriction enzyme-digested genomic DNA followed by Southern blotting and indirect end labeling to visualize the formation and resection of meiotic DSBs [10, 28–30]. Combined with two-dimensional gel electrophoresis, Southern blotting allowed the visualization of DNA cleavage, DNA end resection, and formation of branched DNA recombination intermediates at an artificial hotspot, *HIS4LEU2* [31], yielding more precise measurement of resection lengths ranging 350–1,550 nucleotides (nt) with a mean of ~800 nt [17]. Quantitative but less spatially precise information was provided by restriction enzyme digestion coupled with quantitative PCR (RE-qPCR), measuring the loss of restriction sites as they were converted to ssDNA by resection [32, 33]. These studies provided important insights, but these methods were limited to analyzing a single hotspot at a time, or even just one side of a single hotspot. Moreover, it has so far been impossible to apply these methods in multicellular organisms, where meiosis occurs in a small population of germ cells within a complex reproductive tissue.

The advent of next-generation sequencing revolutionized resection analysis. Single-stranded DNA sequencing (SSDS) after chromatin immunoprecipitation (ChIP) has been applied in various organisms, including plants, mice, and humans, generating genome-wide maps of recombination initiation sites [34–38]. By combining ssDNA coverage maps from DMC1-SSDS with precise mapping of SPO11 cleavage sites from sequencing of SPO11 oligos in mice, resection lengths per DSB end were estimated to range 300–1,800 nt with a mean 894 nt [25]. However, because SSDS samples the distribution of ssDNA bound either by strand exchange proteins (DMC1 or RAD51) or the ssDNA-binding protein RPA, and because these proteins do not cover the entirety of the ssDNA [38], these studies provided only an indirect measurement that was likely an underestimate of true resection tract lengths.

More recently, methods like S1-seq and END-seq provided nucleotide-resolution insights into meiotic resection [16, 20–22, 39]. These methods involve ligation of sequencing adapters to the duplex DNA ends left after digestion of the ssDNA tails with one or more nucleases (Table 1). From this more direct measurement of resection endpoints, resection tract lengths averaged 822 nt in yeast and 1,117 nt in mice, and the effects of various mutants could be studied [16, 20–22].

The ability to measure meiotic DSB resection directly and quantitatively across the genome at nucleotide resolution was an important breakthrough in the field, but one limitation is that the current methods are based on the population average across large numbers of cells. A tool to monitor individual resection progression at the single-cell level would enhance understanding of variation in resection length between individual DSB events.

## 3. Resection initiation by MRX/N

The MRX/N complex (Mre11-Rad50-Xrs2 in *S. cerevisiae*, MRE11-RAD50-NBS1 in mice) plays a crucial role in initiating the resection of meiotic DSBs, removing approximately 300–400 nucleotides from the break site in yeast [16–18]. Sae2 (CtIP in vertebrates) is an essential cofactor required for this resection initiation. While *mre11, rad50,* and *xrs2* null mutants in yeast are viable, similar mutations are lethal in many other organisms, particularly for embryonic development in mice. As a result, the roles of MRX/N in meiotic recombination were initially identified with budding yeast null mutants and later investigated in greater detail using non-null mutations in yeast and other organisms.

### 3.1. Functional domains of the conserved MRX/N complex

**Figure 2.**
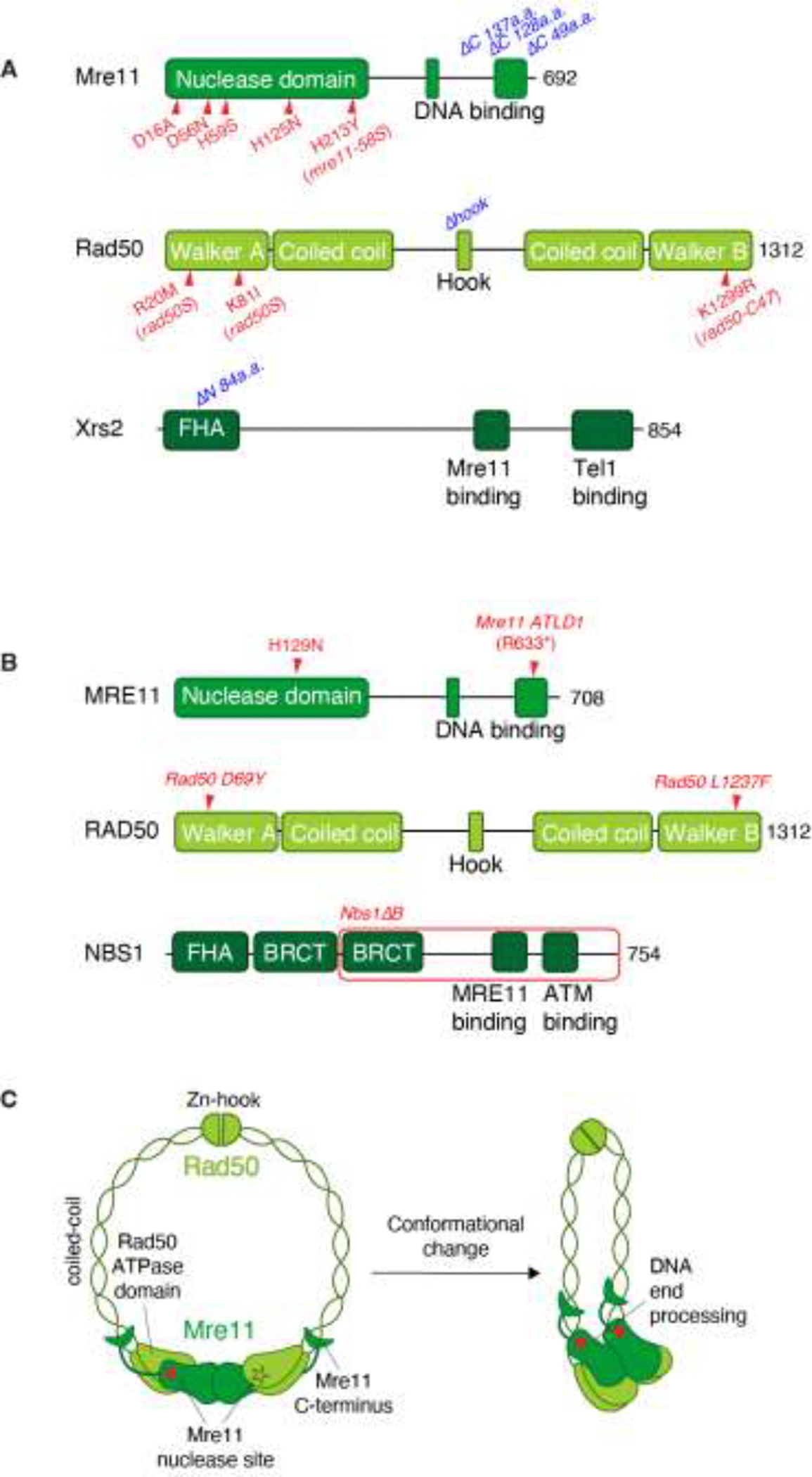
Schematic depiction of functional domains and structural transitions of the MRX/N complex. (A) Functional domains of yeast Mre11, Rad50 and Xrs2 proteins. Non-null mutations defective for meiotic DSB formation (blue) and resection (red) are summarized. Two of nine *rad50S* alleles [10] are marked. (B) Functional domains of human MRE11, RAD50 and NBS1 proteins. Mutations tested for meiotic resection in mice are indicated. (C) A ring-like “resting mode” structure of MR, which undergoes conformational changes into a rod-like “cutting mode” shape [44, 132–135].

Mre11 is a Mn^2+^-dependent single-strand endonuclease and 3’-to-5’ exonuclease in vitro [11, 40, 41] but its endonuclease activities can be strongly promoted in Mg^2+^-only conditions when Sae2 is present along with Rad50 and Xrs2 [42]. The nuclease domain is located at the Mre11 N terminus, followed by DNA-binding domains (Figure 2A,B). Rad50 contains Walker A and B ATPase motifs, separated by a long coiled-coil region and a conserved hinge region within the coiled-coil domain (Figure 2A,B) [43, 44]. Unlike Mre11 and Rad50, Xrs2/NBS1 lacks enzymatic activity but serves as a scaffold through protein-protein interaction motifs [45–48].

Whereas homologs of Mre11 and Rad50 are conserved across all organisms including archaea and even some bacteriophage, Xrs2/NBS1 is thus far found exclusively in eukaryotes [47, 49]. Xrs2/NBS1 plays a key regulatory role by interacting with the C-terminus of Mre11 via its Mre11-interacting domain, a contact reported critical for stimulating endonuclease activity of human MRE11 at protein-blocked DNA ends [50–52]. Xrs2/NBS1 contains an FHA (Forkhead-Associated) domain and, in NBS1, tandem BRCT (BRCA1 C-terminal) domains at its N terminus (Figure 2A,B), which mediate interactions with phosphorylated proteins, including Sae2/CtIP, Lif1, and MCD1 [53–59]. The C-terminal region of Xrs2/NBS1 contains a Tel1/ATM biding motif, deletion of which results in a phenotype similar to *tel1*Δ mutants in yeast, highlighting roles in DNA damage signaling [46].

### 3.2. Essential role of the MRX complex in meiotic DSB formation in budding yeast

The *mre11* mutation was first identified in budding yeast in a screen for mutants defective in meiotic recombination [60]. Virtually identical phenotypes were observed in *rad50* and *xrs2* deletion mutants [10, 61]. This defect was attributed to the absence of meiotic DSBs, revealing that the MRX complex is essential for DSB formation in *S. cerevisiae*. The C-terminal region of Mre11 is strictly required for this function [11, 13, 62]. Interestingly, complex formation between Mre11 and Rad50 may not be necessary for DSB formation, as the *mre11–58S* mutant, which is defective in Rad50 binding, still supports meiotic DSB formation [13, 63]. However, Rad50 dimerization through its hook domain is required [44, 64].

Xrs2 is necessary for the nuclear localization of Mre11 [48], and consistently, the expression of Mre11 fused to a nuclear localization signal can rescue the spore lethality in *xrs2* null mutants [65]. Xrs2 further promotes meiotic DSB formation through its N-terminal region [66]. Although the exact mechanism remains unclear, MRX may facilitate DSB formation through a direct interaction between Mre11 and Spo11 [67, 68].

Notably, the requirement for the MRX complex for meiotic DSB formation is observed only in budding yeast and *Caenorhabditis elegans* [69] but not in other organisms, including other fungi [70–72], *Tetrahymena thermophila* [73], plants [74–77], and possibly mice [22, 78, 79].

### 3.3. Yeast Mre11 nuclease activity: initiating and executing short-range resection

The role of the MRX complex in meiotic DSB resection was further uncovered through non-null mutations (summarized in Figure 2A) that disrupt meiotic DSB repair while preserving other MRX functions. Some of these, termed separation-of-function mutants (*rad50S* or *mre11S)*, form Spo11-induced DSBs but fail to process them [9, 10, 13, 62, 63, 80]. Unlike *rad50* or *mre11* null mutants, these separation-of-function mutants retain nearly normal resistance to methyl methanesulfonate (MMS), they do not exhibit hyper-recombination during mitotic growth, and they cause constitutive upregulation of Tel1 activity [10, 63, 81]. These mutants thus revealed a specific role of MRX complex that is essential for meiotic DSB resection.

Meiotic DSBs are unique in having Spo11 covalently attached at their 5’ ends. Given the knowledge that yeast Rad50 and Mre11 form a complex [82] and share homology with *E. coli* and phage T4 nuclease complexes (SbcC-SbcD and gp46-gp47, respectively [83, 84]), it was proposed that the MRX complex initiates DSB resection by removing Spo11 through its nuclease activity [9]. Consistent with this hypothesis, yeast strains with nuclease-dead *mre11* (*mre11-D16A, D56N* or *H125N)* accumulate unresected DSBs [11–13]. Direct experimental evidence later confirmed that endonucleolytic cleavage releases Spo11 still bound to a short oligo in budding yeast, fission yeast, mouse, and *Arabidopsis thaliana*, mediated by Mre11 where tested [11, 14, 15, 22, 85–88].

Mre11 exhibits single-strand endonuclease and 3’-to-5’ exonuclease activities in vitro [13, 40, 89], yet in vivo, both meiotic and nonmeiotic DSB resection proceeds in the 5’-to-3’ direction [29, 90]. This paradox was resolved by identifying a two-step mechanism: Mre11 first cleaves DNA endonucleolytically, followed by local 3’-to-5’ exonucleolytic processing, which then enables long-range 5’-to-3’ resection by other nucleases such as Exo1 or Dna2-Sgs1 [15, 91]. Notably, in *mre11-H59S* mutants, which retain partial endonuclease but have reduced exonuclease activity, Spo11 oligos are formed but display increased size heterogeneity, supporting a bidirectional resection model [15].

In the absence of Exo1 or its nuclease activity, resection lengths decrease to ~300 nt on average as measured by both Southern blotting and S1-seq [16–18]. This residual resection is considerably further than the length of Spo11 oligos, most of which are less than ~40 nt long [15]. This discrepancy can be resolved by a combination of iterative nicking by MRX-Sae2 and 3′-to-5′ digestion of Spo11 oligos by Mre11, considering that both Southern blotting and S1-seq pick up only those resection-associated strand breaks that are furthest from the DSB end [16]. Experimental support for the ability of MRX to nick iteratively has been provided by both in vitro and in vivo studies of non-meiotic DSB resection [92, 93].

### 3.4. *Sae2/CtIP* and meiotic DNA end resection

Sae2 (also known as Com1) was initially discovered by its requirement for successful meiotic recombination [94, 95]. In *sae2* null mutants, DSBs are formed but remain unresected with Spo11 still covalently attached, similar to the phenotype of *rad50S* and nuclease-dead *mre11* mutants [94–97].

CtBP-interacting protein (CtIP) was originally characterized for its interaction with CtBP, a co-regulator that binds the C terminus of adenovirus E1A [98], and was later found as an ortholog of Sae2 in human and shown to directly interact with the MRN complex and promote DNA end resection [99]. Although Sae2 and CtIP have only limited sequence similarity, their roles in the initiation of DSB resection are widely conserved, with orthologs found in various organisms including fission yeast (Ctp1) and plant (Com1), and shown to function with Mre11 [73, 99–103]. In *C. elegans* and *T. thermophila*, the Sae2 orthologs Com-1/Com1 are essential for meiotic progression, and their loss results in incomplete meiotic DSB repair [73, 102]. Similarly, *com1* mutations in plants lead to sterility due to a failure in meiotic progression [103–105]. In mice, deletion of *Ctip*, like that of components of MRN complex, results in early embryonic lethality [106]. Testis-specific conditional deletion of *Ctip* leads to premature depletion of germ cells [22], so a potential role of CtIP in mammalian meiosis remains to be fully explored.

Sae2/CtIP is extensively phosphorylated by cyclin-dependent kinase (CDK) [107–111] and by DNA damage-dependent kinases Tel1/ATM and Mec1/ATR (ataxia-telangiectasia and Rad3–related) [112–116]. The cell-cycle dependent phosphorylation of Sae2/CtIP by CDK is one of the key mechanisms that restricts extensive DNA end resection (and therefore DSB repair by recombination) to the S and G2 phases of the cell cycle [117]. The phosphorylation of Sae2/CtIP also regulates its multimerization, which is critical for its association with MRX/N complex [114, 118–122]. Physical interaction between CtIP and NBS1 occurs through the FHA and BRCT phosphopeptide-binding domains of NBS1 and the N terminus of CtIP containing putative CDK sites [58]. A crystal structure of the N terminus of fission yeast Nbs1 and a phosphopeptide from the Ctp1 N terminus further supports their phosphorylation-dependent interaction [59].

Phosphorylated Sae2/CtIP stimulates the endonuclease activity of Mre11 in a reconstituted system using short linear duplex DNA with biotin/streptavidin blocks at 5′ and/or 3′ ends [42, 50, 110]. This stimulation depends on the direct interaction between phosphorylated Sae2 and MRX, as well as ATP hydrolysis by Rad50 [110]. Interactions between Sae2/CtIP and the N-terminal region of Xrs2/NBS1 have been reported [58, 59, 115, 119]. However, Xrs2 is dispensable for resection in vegetative cells and is no longer required for successful sporulation when Mre11 is artificially localized to the nucleus [65]. Moreover, Sae2 can promote the endonuclease activity of Mre11 in vitro in the context of MR alone (without Xrs2) [65]. Additional contacts with other regions of the MRX/N complex may also exist [65, 119]. Thus, it remains incompletely understood how Sae2/CtIP interactions contribute to the full regulation of MRX/N during meiotic resection.

Phosphorylated Sae2 also stimulates the 3′-to-5′ exonuclease activity of Mre11 in vitro [92]. This stimulation was reported to be specifically defective with a K1299R mutation in the C-terminal region of Rad50 (referred to as the *rad50-C47* mutation) [123]. Interestingly, the *rad50-C47* mutant showed a number of phenotypes not seen with the exonuclease-defective *mre11-H59S* mutant, including a delay in meiotic progression similar to that in the strong *rad50S* mutant *rad50-K81I,* a partial delay in the appearance of Spo11-oligo complexes suggesting a defect in endonuclease activity in vivo, and appearance of a class of Spo11 oligos with a 10-bp periodicity in oligo length similar to that previously reported for instances where multiple Spo11 complexes cut the same DNA molecule in close proximity (so-called Spo11 double cutting) [15, 123–125]. It is currently unclear why a defect in Sae2-stimulated Mre11 exonuclease (*rad50-C47*) would give a phenotype so different from constitutive exonuclease attenuation (*mre11-H59S*), but a contributing factor may be the moderate *rad50S*-like properties of *rad50-C47* that include reduced endonuclease activity on Ku-blocked DNA ends in vitro and enhanced Tel1 activity [123].

It has also been reported that Sae2/CtIP itself exhibits nuclease activity on branched DNA structures [126–128], but this remains controversial [42, 118, 119, 129]. When Sae2/CtIP was mutated to destroy the reported nuclease activity without eliminating stimulation of Mre11, cells manifested mitotic recombination defects, hypersensitivity to DNA damaging agents and reduced sporulation [130]. If Sae2/CtIP does indeed have nuclease activity, it appears to be biochemically separable from its role in stimulating Mre11 endonuclease activity [130, 131]. It remains to be clarified whether Sae2/CtIP exhibits nuclease activity in vivo and, if so, how it contributes to Spo11 removal from DSB ends.

### 3.5. Structural dynamics of the MRX/N complex

The MRX/N complex comprises two copies each of Mre11 and Rad50, along with one or two copies of Xrs2/NBS1, and undergoes conformational changes upon DNA binding from a resting to an active cutting mode (Figure 2C) [44, 132–137]. A 2:2:2 stoichiometry was initially proposed, but recent high-resolution cryo-EM analysis strongly supports a 2:2:1 configuration for the MRX/N complex, in which heterotetrametric M_2_R_2_ binds to a single NBS1 asymmetrically [135]. MRX/N complexes have a globular head domain containing Mre11 and the ATPase domains of Rad50, from which the coiled-coil domains of Rad50 extend and terminate in a zinc hook that mediates dimerization and DNA tethering [138, 139]. DNA binding is mediated primarily by the head domain, though both the Rad50 coiled coils and Xrs2/NBS1 contribute to DNA engagement and complex stabilization [135, 136, 140].

Cryo-EM studies of the *E. coli* homolog of the Mre11-Rad50 complex (SbcC-SbcD) have provided key insights into the complex’s structural dynamics [132]. Upon DNA binding, the Rad50 coiled-coil domains close to form a narrow clamp around a single DNA duplex, triggering the relocation of the Mre11 dimer from the bottom to the side of the complex. This structural shift allows Mre11 to engage blocked DNA ends and activates its endonuclease function. The transition to this “cutting mode” is stabilized by an interaction between the N-terminal “fastener loop” of bacterial Mre11 and the nucleotide-binding domain of Rad50 [132]. In budding yeast, this interaction surface is conserved and is where *rad50S* mutations cluster [10]. It was proposed that the function of the bacterial Mre11 fastener loop has been replaced by Sae2/CtIP in eukaryotes, which is essential for activating the endonucleolytic activity of MRX/N. This model helps explain why complexes containing Rad50S mutant proteins are biochemically unresponsive to Sae2 [132]. Additionally, when Rad50 is bound to ATP, it partially blocks the nuclease active site of Mre11, inhibiting its 3’-to-5’ exonuclease activity [132, 141]. Upon ATP hydrolysis by Rad50, a conformational change exposes the active site of Mre11, enabling endonucleolytic cleavage of blocked DNA ends [14, 42, 50, 142, 143].

This conformational control provides a potential explanation for the phenotypes of *S. cerevisiae rad50S* mutants that are similar to nuclease-dead *mre11* mutants and *sae2* null mutants [10, 94, 95, 144]. Notably, *rad50S* mutations cluster near the nucleotide-binding domains of Rad50, although they exhibit distinct phenotypes from *rad50* null or ATP hydrolysis-defective mutants, thereby suggesting that these mutations may impair an interaction site for a meiosis-critical protein [10]. Seven of the nine *rad50S* mutations are located adjacent to the proposed DNA binding groove, and none of *rad50S* mutations are directly involved in ATP binding, further strengthening their structural roles rather than roles in the enzymatic activity of Rad50 [132, 145]. The conformational activation of MRX/N is further modulated by Sae2/CtIP, which alleviates Rad50-mediated inhibition of Mre11 and enhances DNA end resection [42, 50]. Consistently, at least one *rad50S* mutation (K81I) also disrupts the physical interaction between MRX and Sae2 [92, 110], providing a functional basis that explains the indistinguishable phenotypes observed in *rad50S*, *mre11-nd* and *sae2* null yeast cells.

### 3.6. The MRN complex and mammalian meiotic DSB processing

Studying MRN in mouse meiosis is challenging due to embryonic lethality resulting from the knockout of any component of the complex [146–148]. To circumvent this, recent studies have focused on targeted gene deletion in the germline and characterization of hypomorphic MRN mutations that were developed based on mutations in human genome instability syndromes [149–155] (Figure 2B).

Conditional deletion of *Mre11* in spermatogonia caused accumulation of unresected DSBs [22]. By contrast, conditional *Rad50* deletion caused only partial resection defects [78], possibly reflecting limitations of conditional deletion approaches. A mouse model of a *rad50S* allele from *S. cerevisiae* (mouse *Rad50*^*K22M*^ [156]) did not display any resection defects [22], but this result is likely uninformative about MRN roles in resection because this mutation is based on a relatively weak *rad50S* allele (*rad50-R20M* in yeast [10]). A *Ctip* conditional knockout was also uninformative because it resulted in death of spermatogonia before meiotic entry [22].

Taken together, these findings indicate that the MRN complex is essential for resection initiation but may not be required for DSB formation in mammalian meiosis. Dispensability of MRN in DSB formation is also suggested by the finding that DSBs are formed in *Mre11*^*ATLD1*^ mice, which model ataxia telangiectasia-like disorder [152, 157]. The *Mre11*^*ATLD1*^ mutation removes the MRE11 C-terminal region (Figure 2B), which is essential for DSB formation in yeast [13].

Resection was initiated but resection tract lengths were substantially shortened in mice homozygous for *Mre11*^*ATLD1*^ and *Nbs1*^Δ*B*^ mutations [22]. *Nbs1*^Δ*B*^ models Nijmegen breakage syndrome [153]. Reductions in resection lengths were also observed in *Rad50* mutants modeling recurrent human cancer mutations in Walker A and B ATPase motifs, and in mice conditionally expressing a nuclease-defective allele of *Mre11* (*Mre11*^*H129N*^) [22]. Most of these mutants had substantially shorter resection than in *Exo1* nuclease-dead or *Exo1-null* mutants [20, 21].

**Figure 3.**
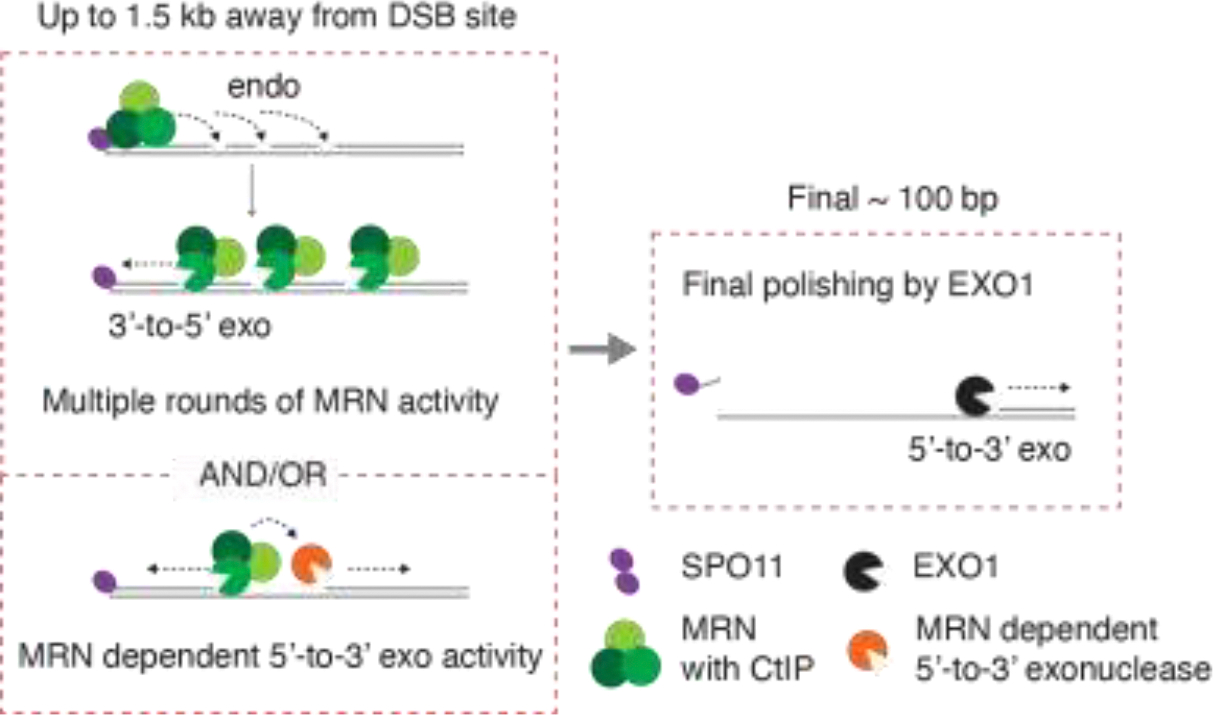
Possible resection mechanisms in mammalian meiosis, where MRN is needed for the majority of 5’ strand degradation, either through multiple rounds of its own activity or by promoting an unknown MRN-dependent exonuclease that is distinct from EXO1.

Seeing such shortened resection was surprising because the simple extrapolation from the yeast two-step resection model predicted that long-range nuclease(s) should be able to carry out the full extent of resection as long as resection had initiated. This MRN contribution to resection length appears parallel to EXO1 function, as *Nbs1*^Δ*B*^ *Exo1* nuclease-dead double mutants exhibit an additive reduction in resection length [22, 158]. These findings suggest that MRN-dependent activity drives the majority of resection in mouse meiosis, while EXO1 primarily refines the final part of resection. Plausible scenarios include MRE11 endo- and exonuclease activities working iteratively to extend resection further than occurs in yeast, or MRN promoting an unknown nuclease other than EXO1 (Figure 3, see also Section 4.2).

## 4. Long-range resection: extension by Exo1

### 4.1. Exo1 drives long-range resection in yeast meiosis

Mitotic long-range resection involves either or both Exo1/EXO1 and Dna2-Sgs1 (DNA2-BLM or DNA2-WRN) nucleases [18, 91, 159–161]. However, in yeast meiosis, the *exo1* nuclease-dead mutant (*exo1-nd*) reduces resection length by ~50%, but loss of Dna2-Sgs1 activity has no detectable impact [16, 17]. The contribution of Dna2-Sgs1 is detectable only at late time-points for the hyper-resection that occurs in recombination-deficient *dmc1*Δ mutants [111]. Whether Dna2-Sgs1 homologs are similarly dispensable in mammalian meiosis has not yet been addressed.

Early experiments in yeast showed that mitotic long-range resection generates extensive ssDNA overhangs [18, 162], with lengths far exceeding meiotic resection (e.g., from 2 to over 80 kb [18, 163]). However, these estimates may be inflated due to pathological consequences of DNA repair defects. Most studies have been conducted in engineered backgrounds where artificially induced DSBs (typically by HO or I-SceI) cannot be repaired via the canonical homologous recombination pathway using the sister chromatid as a template. When a sister chromatid template is available, resection tracts are shorter (~1 kb) [164], comparable to meiotic resection length [16, 17]. Thus, mitotic and meiotic resection lengths may be fairly similar under normal conditions.

### 4.2. Conservation and divergence of Exo1 in meiotic resection

Exo1 drives meiotic resection in various species, including the multicellular nematode *C. elegans* and the unicellular protozoa *T. thermophila*, as assessed cytologically by formation of chromosome-associated foci of DSB repair factors [165, 166]. In mouse spermatocytes, the absence of EXO1 protein or of just its nuclease activity reduces the mean resection length by only ~10% [20, 21]. This contrast with the more pronounced reduction in budding yeast suggests that another nuclease(s) may contribute to resection in mammalian meiosis, either MRN itself or another MRN-dependent nuclease (see Section 3.6).

Notably, this observation challenges the traditional binary classification of resection into “short-range” and “long-range” based on length, indicating the need for a more mechanistic definition of resection pathways. Further investigation is required to uncover divergent aspects of meiotic DSB resection in mammals, potentially including the identification of additional nuclease(s) beyond MRN and EXO1 that may function in germ cells or the discovery of regulatory mechanisms that enhance MRN activity in mammals compared to yeast.

### 4.3. Exo1 structure, function and regulation

During efforts to determine whether nucleases promote recombination, a nuclease activity was identified in protein extracts of *Schizosaccharomyces pombe* undergoing meiosis [167]. This nuclease, termed exonuclease-1 (Exo1) degrades only the 5’ strand, producing 3’-ended single-stranded DNA (ssDNA) tails [167]. The *exo1*Δ mutations reduce exonuclease activity in protein extracts from *S. pombe* and *S. cerevisiae* [168–171]. *EXO1* transcription increases during meiosis in *S. pombe*, *S. cerevisiae*, the female germline of *Drosophila melanogaster* (*EXO1* homolog *tosca*), and testes of *Mus musculus* and *Homo sapiens* [171–175]. Later studies demonstrated that *S. cerevisiae* Exo1 also participates (independent of its nuclease activity) in crossover resolution by the MutLγ complex (Mlh1-Mlh3), RFC-PCNA, and Cdc5 [17, 176–179].

#### 4.3.1 Exo1 domain structure

Exo1 is a 5’-to-3’ strand-specific double-stranded DNA (dsDNA) exonuclease, which belongs to the Rad2/XPG family of nucleases [167, 168, 180]. Eukaryotic Exo1 is divided into two functional halves: an N-terminal nuclease catalytic domain and a C-terminal intrinsically disordered region that regulates activity through post-translational modifications and that interacts with Cdc5, Msh2, and Mlh1 [179, 181, 182].

**Figure 4.**
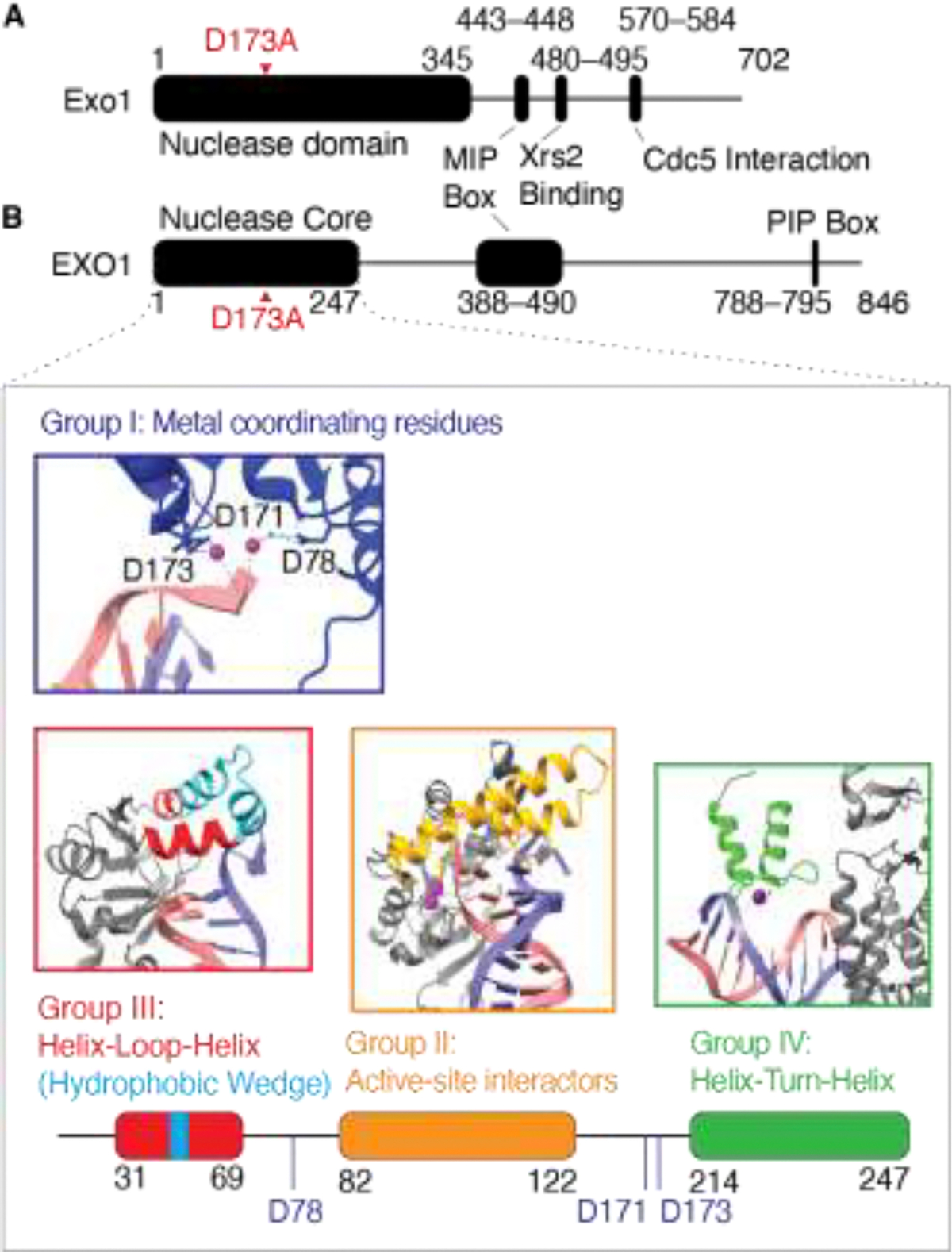
Exo1 domain structure. (A) Domain structure of *S. cerevisiae* Exo1. Mutation of a metal-coordinating residue (red arrow) abolishes nuclease activity. The C-terminal half includes an Mlh1-interacting peptide (MIP) box, an Xrs2-binding motif, and a Cdc5 interaction motif. (B) Domain structure of human EXO1. The N-terminal half contains the nuclease core, which can be subdivided into four groups: Group I (metal-coordinating residues, blue: D78, D171, and D173), Group II (active-site interactors, orange), Group III (helix-loop-helix, red, with the hydrophobic wedge in cyan), and Group IV (helix-turn-helix, green). The C-terminal half contains the MIP box and PCNA-interacting Protein (PIP) box.

The N-terminal catalytic domain can be further divided into four groups of functional amino acid residues (Figure 4). The first group comprises three metal-coordinating residues that hydrolyze the DNA phosphodiester backbone [183, 184]. A mutation of the highly conserved metal-coordinating aspartate (D173A, also known as *exo1-nd*) disrupts nuclease activity but maintains DNA binding capacity both in vitro and in vivo [17, 170, 185, 186]. The second group stabilizes the scissile bond adjacent to the catalytic metal forming the Exo1 active site and helps in unwinding the DNA duplex and positioning the DNA strand to be cleaved [184]. The third group forms a helix-loop-helix and contains a hydrophobic domain that induces a sharp turn at the ssDNA-dsDNA junction. The fourth group is a helix-turn-helix DNA-binding domain that stabilizes Exo1 on DNA and facilitates the movement of the protein along the DNA backbone.

Mutations in the first group of metal-coordinating or second group of active-site residues lead to defects in nuclease activity but do not impair meiotic crossover formation [17, 32, 183]. In contrast, mutations in the fourth group disrupt Exo1 DNA binding, resulting in loss of nuclease activity and meiotic crossovers, mimicking the phenotypes of *exo1*Δ and *mlh1*Δ mutants [183, 187] and highlighting that nuclease and crossover-promoting activities are separable.

The C-terminal half of Exo1 is mostly intrinsically disordered and poorly conserved among eukaryotes, except for the Mlh1-interacting peptide (MIP) box [188, 189]. In addition, an *S. cerevisiae* Exo1 region (480–495)—conserved within the yeast subphylum *Saccharomycotina*—is essential for interaction with Xrs2; its deletion reduces meiotic resection tracts by ~30% (Hasan Alnaser and Bin Hu, unpublished results). Another region (R570–Y584)—also conserved within *Saccharomycotina*—interacts with Cdc5, a polo-like kinase critical for crossover resolution [179, 190]. An *exo1* mutant deficient in Cdc5 interaction shows reduced crossovers [179]. Thus, the Exo1 C-terminal domain plays temporally distinct regulatory roles: first, it recruits Exo1 to DSB sites through interaction with Xrs2, and later it promotes crossover resolution through recruitment of Mlh1 and Cdc5.

#### 4.3.2 Exo1 regulation

Yeast Exo1 undergoes various post-translational modifications, including phosphorylation and ubiquitination [179, 191–193]. Similarly, human EXO1 is subject to phosphorylation, ubiquitination, and SUMOylation in response to DNA damage in somatic cells [194–196].

During yeast meiosis, Exo1 is phosphorylated at serine/threonine (S/T) residues in its C-terminal region [179, 191]. In the *rad51-II3A dmc1-II2A* double mutant, where recombinases assemble at resected DSBs but DSB repair is blocked, suppressing the activity of Mek1 kinase (a meiosis-specific paralog of the Rad53 checkpoint kinase) reduces Exo1 phosphorylation and leads to hyper-resection of DSBs [191]. Thus, Exo1 phosphorylation, at least in part, acts to suppress aberrant hyper-resection.

Similar to Exo1 regulation in yeast meiosis, human EXO1 is also regulated by phosphorylation in somatic cells. Initially, CDK1/2 phosphorylates EXO1 to activate its nuclease activity, followed by phosphorylation by ATM/ATR kinases, which triggers ubiquitination and SUMOylation, leading to EXO1 degradation and preventing excessive resection [194, 196, 197]. Likewise, a recent study in vegetative yeast found that Exo1 undergoes ubiquitination, suggesting that long-range resection may be downregulated through Exo1 degradation [192].

Some meiotic phosphorylation sites on *S. cerevisiae* Exo1 match consensus sequences of Tel1/Mec1 (S/T-Q), Mek1 (RxxS/T), and Cdc28 (S/T-P) [198–200]. Investigating whether Exo1 is ubiquitinated during meiosis, as well as the individual roles of its many phosphorylation sites, will further elucidate the mechanisms regulating Exo1 activity during meiosis.

## 5. Tel1/ATM and Mec1/ATR function in meiotic DSB resection

In response to Spo11-induced DSBs, the DNA damage response kinases Tel1/ATM and Mec1/ATR activate the meiotic checkpoint network [201, 202], which in turn regulates resection.

### 5.1. Recruitment and activation

Tel1/ATM is activated by directly interacting with the MRX/N complex [203]. In budding yeast, Tel1 is activated by the MRX complex upon Spo11-induced DSB formation [81]. The C-terminal domain of Xrs2 recruits Tel1 to chromosome axis sites and Spo11 DSB hotspots [204]. Mec1/ATR, on the other hand, is activated by the formation of RPA-coated ssDNA via Rad24/ATRIP and the 9-1-1 complex (RAD9-RAD1-HUS1; Rad17-Mec3-Ddc1 in budding yeast) at ssDNA/dsDNA junctions [205, 206].

Both Mec1/ATR and Tel1/ATM primarily phosphorylate serine or threonine residues followed by glutamine (SQ/TQ motifs) [199, 207]. They phosphorylate multiple factors, including subunits of the MRX/N complex [208–210], and CtIP/Sae2 [112–116].

### 5.2. Control of resection initiation and extension by Tel1/ATM and Mec1/ATR

In budding yeast, unresected meiotic DSB ends activate Tel1-mediated phosphorylation of Sae2 to facilitate resection initiation, which in turn activates Mec1, also facilitating Sae2 phosphorylation [112, 116]. Mec1 appears to also phosphorylate Sae2 and initiate meiotic resection independently of Tel1 [112], implying that its role in resection is not strictly Tel1-dependent. However, in other yeast strain background, the Sae2 phosphorylation by Mec1/Tel1 is not strictly required to initiate meiotic resection [211]. Furthermore, in vegetative cells, this phosphorylation promotes resection extension—rather than initiation—by attenuating DNA damage signaling, which otherwise inhibits Exo1 recruitment [211]. Notably, S1-seq analysis in *tel1*Δ detected a mix of both unresected DSB ends and shorter resection tracts [16], suggesting that Tel1 is involved in both resection initiation and extension.

*Atm*-null mice also exhibit a mix of both resected and unresected DSB ends, with resection tracts varying widely in length, spanning from much shorter to substantially longer than wild type [20, 21]. Reducing *Spo11* gene dosage (*Spo11*^+/−^) eliminates the hyper-resected subpopulation without affecting the accumulation of hypo-resected and unresected DSBs in *Atm*^−/−^, resulting in a phenotype that resembles observations in *tel1*Δ yeast [16, 20, 21].

ATM/Tel1 also regulates DSB numbers through a negative feedback mechanism that suppresses excessive DSB formation (reviewed in [212–214]). Notably, the degree of DSB suppression differs between yeast (about twofold) and mice (about tenfold) [215–220]. Because *Spo11* heterozygosity in mice attenuates both the increased DSB formation and hyper-resection in the *Atm*^−/−^ background [21, 215], the hyper-resection may itself be a consequence of massively increased DSBs.

Intriguingly, Mec1 and the 9-1-1 complex limit resection tract length in *S. cerevisiae* [221, 222] (see also Section 7). Thus, Mec1 functions as both a promoter of resection initiation [112], and an inhibitor of excessive resection.

## 6. Chromatin remodeling facilitates efficient meiotic resection

**Figure 5.**
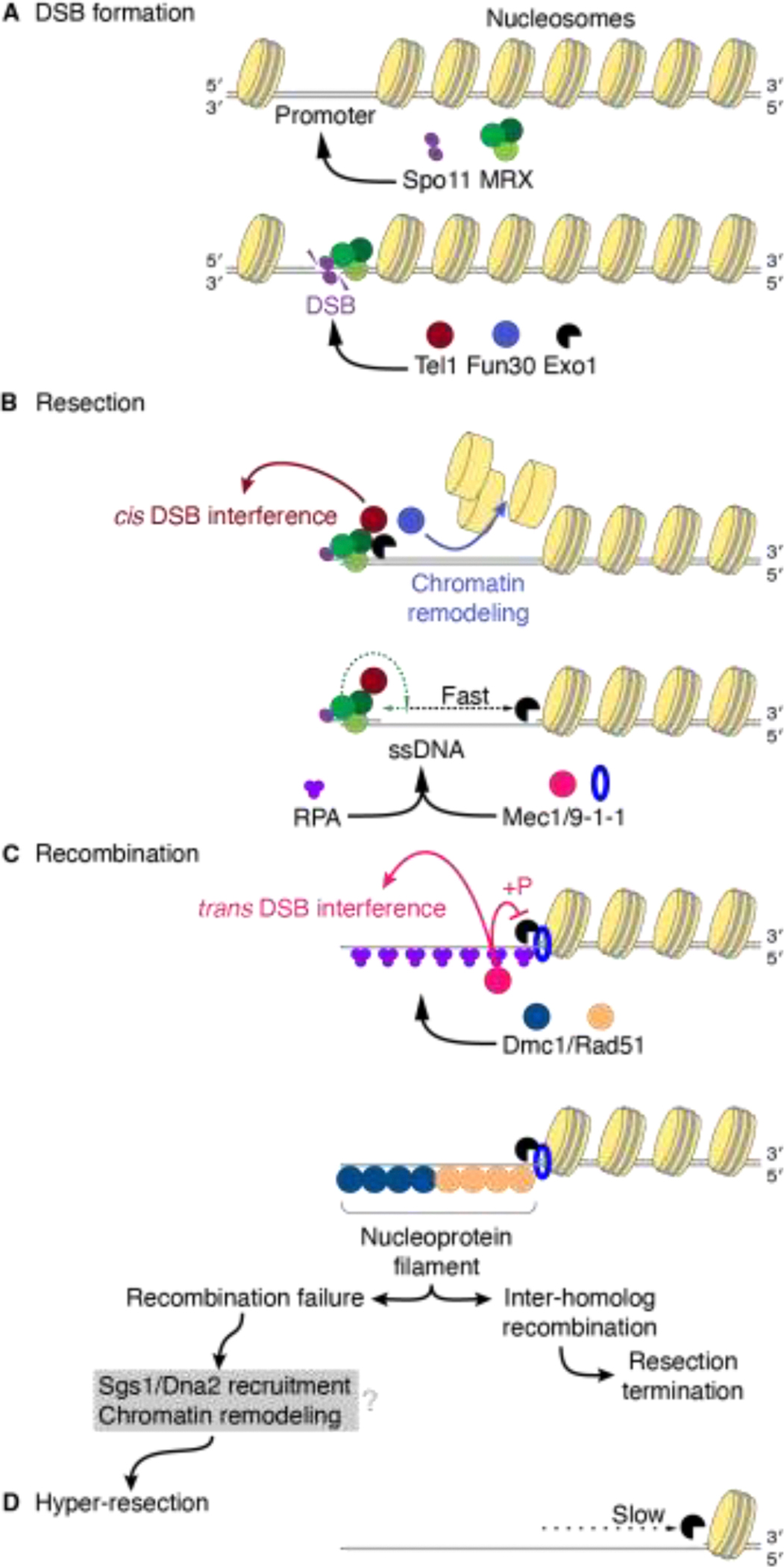
Multilayered mechanisms coordinating DSB formation, resection and recombination in yeast meiosis. (A) DSB Formation. Spo11, recruited with the MRX complex to the promoter NDR, introduces a DSB that, in turn, recruits Tel1 and Fun30. (B) Initiation and extension of resection. Following DSB formation, Fun30 remodels chromatin to promote both resection initiation by MRX and Sae2 (not shown), and resection extension by Exo1. The resulting 3’ ssDNA tail is bound by RPA, Mec1, and the 9-1-1 complex. Tel1 activates DSB interference in *cis*, ensuring that chromatin remodeling occurs preferentially where further DSB formation has been suppressed. (C) Recombination. Mec1 and the 9-1-1 complex activate DSB interference in *trans* and possibly downregulate Exo1 to limit excessive resection. RPA is replaced by Dmc1 and Rad51 recombinases, forming a nucleoprotein filament that carries out homologous recombination, which terminates resection. (D) Hyper-resection. In recombination-deficient mutants, resection continues at a slower rate compared to normal resection, leading to excessive DNA degradation. This hyper-resection possibly involves Sgs1/Dna2 recruitment and chromatin remodeling.

Chromatin structure plays vital roles throughout the formation, processing, and repair of DSBs during meiosis [5, 213, 223]. Meiotic DSB hotspots in budding yeast and mice coincide with nucleosome-depleted regions (NDRs) (Figure 5A), such as transcription promoters in yeast or PRDM9 binding sites in mammals [25, 26, 224–227]. However, meiotic resection endpoints exceed these NDRs and often overlap with the regions where nucleosomes are positioned [16]. Nucleosomes impede MRX/Sae2 and Exo1 activities in vitro and resection in vegetative cells [93, 143, 228]. Given that efficient resection in vegetative cells requires multiple chromatin remodelers (e.g., RSC, INO80, and Fun30) [229], it has been proposed that meiotic resection also requires nucleosome remodeling on broken chromatids [16].

Indeed, Fun30 is critical for meiotic resection in *S. cerevisiae*, but other chromatin remodelers tested (INO80, SWR1) played no detectable role [230] (Figure 5B). Recruited in response to DSB formation, Fun30 likely remodels nucleosomes to promote the initial nicking by MRX-Sae2 and subsequent exonucleolytic processing by Exo1, thereby defining resection endpoints. Furthermore, the *exo1-nd fun30*Δ double mutant exhibits extremely short resection and impaired recombination, likely due to defects in the establishment of interhomolog bias [230]. SMARCAD1 (the mammalian Fun30 ortholog) promotes resection in somatic cells [231, 232], but remains to be evaluated in meiosis.

In most mammals, the DNA binding protein PRDM9 marks meiotic hotspots through a combination of histone modifications [37, 233–236]. The chromatin remodeler HELLS is then recruited and modifies the chromatin to facilitate DSB formation and subsequent repair [237, 238]. HELLS promotes DSB resection in somatic cells [239], but whether it does so during meiosis remains to be investigated. It also remains to be evaluated whether meiotic resection depends on other chromatin remodelers that influence resection in non-meiotic contexts, such as INO80 [240–242].

## 7. Hyper-resection: a distinct mode further extending resection tracts

The length of meiotic resection is also regulated by the subsequent recombination. After resection, the 3’ ssDNA tails are initially coated by RPA, which is then replaced by RecA homologs that mediate homology search and strand invasion—Dmc1 (meiosis-specific) and Rad51 [243] (Figure 5C). In the absence of Dmc1 and/or Rad51, recombination fails, leaving unrepaired resected DSB ends that activate a DNA damage/recombination checkpoint that prolongs prophase I [201, 244]. Interestingly, recombination-deficient mutants in both yeast and mice also exhibit hyper-resection of DSBs—excessive degradation of the 5’ strand [16, 20, 21, 245–247] (Figure 5D).

Hyper-resection in recombination-deficient mutants differs from resection in recombination-proficient cells. For example, a minor contribution from Sgs1 and Dna2 to hyper-resection has been reported [111]. Moreover, hyper-resection in *dmc1* null budding yeast proceeds much more slowly (0.19 kb/hour) than normal meiotic resection (>16 kb/hour) or the resection of irreparable breaks in vegetative cells (~4 kb/hour) [16, 18, 162, 248]. Thus, this hyper-resection appears to operate in a secondary, more conservative fashion. The restraint on hyper-resection possibly prevents deleterious effects of having overly long ssDNA at DSB ends. Given its dependence on Exo1 [175], hyper-resection may also involve chromatin remodeling, although the role of Fun30 in this context remains to be tested.

The mechanism triggering hyper-resection remains poorly understood. Hyper-resection has usually been detected by Southern blotting as faster-migrating, more smeared DSB signals that appear at late time points during prolonged or arrested prophase in recombination-deficient mutants, such as *dmc1*Δ or *dmc1*Δ *rad51*Δ [245, 249, 250]. While this approach lacks the resolution to precisely quantify resection lengths, subsequent molecular characterization has provided important insights into its regulation.

Hyper-resection appears to be associated with profound DSB repair deficiency, as it is less apparent or even undetectable in *rad51*Δ or *hed1*Δ *dmc1*Δ mutants where DSB repair is partially or extensively permitted via inter-sister recombination [247, 249, 251]. Moreover, hyper-resection is observed in many recombination-deficient backgrounds, including mutants defective in efficient Dmc1 nucleoprotein filament formation (*mei5*Δ, *sae3*Δ, *rad54*Δ *rdh54*Δ), strand exchange (*hop2*Δ and *mnd1*Δ) [249, 252, 253], or second-end capture (*rad52* and *rfa1* mutants) [254, 255]. Interestingly, meiotic cohesin mutants (*rec8*Δ, *rec8*Δ::*pREC8-SCC1*) also exhibit hyper-resection concomitant with repair defects [256, 257].

On the other hand, when recombinational repair is slowed down—either due to the absence of homologous chromosomes or sister chromatids—DSBs persist for extended periods but do not exhibit obvious hyper-resection [258, 259]. Furthermore, among mutants of the ZMM group of proteins that bind recombination intermediates and promote crossover formation, only *mer3*Δ displays hyper-resection [260], while other *zmm* mutants (*zip1*Δ, *zip3*Δ, and *msh5*Δ) do not [261, 262].

Thus, although exceptions exist, DSB ends that fail to mature into recombination intermediates often undergo hyper-resection during prolonged prophase. However, these findings should be re-evaluated using quantitative approaches to precisely measure resection length. Furthermore, the biological significance of hyper-resection remains unclear: it could represent either a programmed backup mechanism to resolve persistent DSBs or a non-physiological (pathogenic) consequence of catastrophic defects specific to mutants. A central unanswered question is whether hyper-resection can occur in wild-type cells in response to a transient recombination problem.

In contrast to the late-occurring hyper-resection observed in recombination-deficient mutants, 9-1-1 complex and Mec1 mutants (*rad17*Δ, *rad24*Δ, *pCLB2-MEC1*) exhibit hyper-resected DSB ends as soon as the breaks become detectable, even in the absence of additional mutations that impair recombination [221, 222]. Furthermore, these mutations as well as Mek1 suppression exacerbate hyper-resection in recombination-deficient mutant backgrounds [191, 221, 222]. Together, these findings suggest that the 9-1-1 complex and Mec1 negatively regulate resection, possibly through Mek1 activation (see also Sections 4.3.2 and 5; Figure 5C).

Thus, meiotic resection operates through two distinct modes: (1) a conventional mode that enables rapid resection to generate 3’ ssDNA tails of sufficient length for most DSBs to efficiently recombine with homologs; and (2) a secondary mode that slowly extends resection when DSBs persist. In both cases, Mec1—possibly via Mek1—restricts resection tract length [191, 221, 222], potentially to mitigate risks associated with excessive ssDNA accumulation and open chromatin.

## 8. Spatiotemporal coordination of resection and recombination in meiosis

Regulatory linkages ensure that recombination initiation processes occur in the correct sequence. For example, DSB formation activates negative DSB feedback loops that could inhibit additional Spo11 complexes from cleaving the newly opened chromatin generated by Fun30 (Figure 5B,C). In addition to DSB sites, Fun30 is recruited to axis-associated regions in response to DSB formation, suggesting that Fun30-mediated remodeling operates within the context of the tethered loop-axis complex, which includes unbroken chromatids [230]. Consequently, post-DSB remodeling by Fun30 may occur elsewhere on top of the immediate vicinity of the DSB, potentially facilitating recombination processes in addition to promoting resection. However, such NDRs could increase the risk of excessive DSB formation within chromosome domains that have already been cleaved.

This potential risk is likely mitigated by DSB feedback mechanisms involving DNA damage checkpoint kinases, Tel1 and Mec1, which are recruited to DSB sites following cleavage and resection, respectively. Activated Tel1 and Mec1 inhibit Spo11 from breaking nearby hotspots, in cis and trans, respectively, through a process known as DSB interference [216–219]. DSB interference spreads across large genomic regions—e.g., Tel1-mediated interference can cover ~70–100 kb—effectively silencing Spo11 activity in the regions susceptible to Fun30-mediated post-DSB remodeling. Thus, this coordinated regulation spatially restricts chromatin remodeling to those regions that have lost DSB competency, thereby temporally partitioning DSB formation and chromatin remodeling at the chromosome domain level.

Additionally, the involvement of the MRX complex in forming DSBs in budding yeast and *C. elegans* may be advantageous because it enables subsequent steps—such as endonucleolytic resection initiation by Mre11, checkpoint signaling via Tel1 recruitment, and Exo1 recruitment—to be triggered immediately after DSB formation rather than waiting for MRX recruitment after the DSB is formed. Fun30 probably binds to DSB sites at this initial stage, as its recruitment is independent of nicking by MRX-Sae2 [230], although the molecular mechanism behind Fun30 recruitment remains unclear. Exo1 is also recruited in a DSB-dependent manner (Hasan Alnaser and Bin Hu, unpublished results) and appears to persist (or to reassociate) after resection, contributing to crossover formation (see Section 4.3). By employing a group of multifunctional proteins to deliver essential factors to the right place at the right time, various DNA processes—from DSB formation to resection and recombination—proceed seamlessly and in the correct sequence.

These regulatory pathways ensure precise control of meiotic DSB resection, promoting accurate recombination while minimizing risks of ectopic recombination or excessive open chromatin configuration. This understanding enhances our knowledge of both meiotic recombination and broader DNA repair mechanisms crucial for genome integrity.

## Figures and Tables

**Table 1.** Methods for measuring meiotic DSB resection

Method	Description	Applied organism	Targets	Ref
Native/Denaturing 2D-gel Southern Blotting	Genomic DNA are digested by restriction enzymes (targeting outside of resected portion of DNA) to fragment into a smaller size suitable for electrophoresis. Fragmented DNA samples are first subjected to electrophoresis under native conditions to separate DNA molecules based on their size and conformation. Then, DNA samples are further separated only based on their size in a perpendicular direction under the denaturing condition. The separated DNA fragments are transferred onto a membrane and hybridized with labeled probes specific to the DNA sequence of interest.	Yeast	Single hotspot	[17, 263]
RE-qPCR	Genomic DNA samples are digested with restriction enzymes that target the resected part of DNA. Quantitative PCR (qPCR) is then performed using primers flanking the cut site to quantify the amount of ssDNA, which cannot be cut by the restriction enzymes. The relative proportions of ssDNA and dsDNA in the sample can be calculated at a certain distance from the target DSB site, but only as defined by the locations of the restriction sites tested.	Yeast	Single hotspot	[32, 33]
DMC1-SSDS plus SPO11-oligo sequencing	DMC1 (a meiosis-specific recombinase)-bound DNA fragments are captured by ChIP, then a sequencing library is generated after a brief heat denaturation to facilitate hairpin formation by ssDNAs. By using the hairpin-mediated adaptor ligation strategy, sequencing reads from ssDNA are enriched and further processed computationally. Combining SSDS maps with the precise mapping of SPO11 cleavage sites by SPO11-oligo sequencing allows a computational reconstruction of the likely distribution of resection tract lengths.	Mice	Genome-wide	[25, 36]
S1-seq, END-seq	ssDNA at resected DSBs is digested by single-strand specific nucleases, such as S1 nuclease or a cocktail of *Escherichia coli* exonuclease T and exonuclease VII, and the resulting blunted DNA products are captured by ligation to sequencing adapters and sequenced.	Yeast, mice	Genome-wide	[16, 20, 21]
